# A Global Call to Action on Antimicrobial Resistance at the UN General Assembly

**DOI:** 10.3390/antibiotics13100915

**Published:** 2024-09-24

**Authors:** Albert Figueras

**Affiliations:** Independent Researcher, Santos 11045-101, SP, Brazil; albert.figueras@gmail.com

As the United Nations General Assembly prepares to convene its second high-level meeting on antimicrobial resistance (AMR) in September 2024, it is crucial to reflect on the progress made since the first meeting in 2016 and the urgent need for immediate action. Eight years ago, world leaders recognized AMR as a fundamental menace to human health, development, and security. However, today, AMR remains one of the top ten global public health threats facing humanity, contributing to 5 million deaths per year [1].

The 2016 UN declaration highlighted the multifaceted nature of AMR, acknowledging that its primary drivers include the inappropriate use of antimicrobials in the human health, animal health, food, agriculture, and aquaculture sectors [2]. This recognition of the One Health approach—the interconnectedness of human, animal, and environmental health—remains central to effectively addressing the AMR crisis.

Despite the commitments made in 2016, progress since then has been insufficient. The World Health Organization’s 2024 resolution on AMR underscores this stagnation, urging Member States to accelerate national and global responses [3]. This sense of urgency is well founded, as a World Bank report suggested that, if left unchecked, by 2050, AMR could result in economic damage on par with the 2008 financial crisis [4].

As we approach the 2024 high-level meeting, world leaders must recommit to concrete, coordinated action. The upcoming meeting’s theme, "investing in the present and securing our future together", aptly captures the dual imperatives of immediate action and long-term strategy [5]. It has been repeatedly emphasized that key actions should include strengthening global surveillance systems for AMR and antimicrobial use; implementing and enforcing regulations on antimicrobial use in healthcare and agriculture; investing in water and sanitation infrastructure to reduce environmental contamination; supporting research into new antimicrobials, alternative therapies, and rapid diagnostics; and promoting public awareness and education on proper antimicrobial use.

However, antimicrobial use in farms remains high, dispensing without prescriptions is widespread, and water and sewage are not routinely tested. Perhaps part of the problem lies in the foundation of the proposed interventions. On the one hand, we keep focusing campaigns and slogans on words such as “antibiotics” or “antimicrobials”, but are we sure that all audiences know the exact meaning of these terms? On the other hand, perhaps the problems being experienced are not only related to knowledge but also to the willingness to change one’s behavior or the economic consequences of behaving differently. So, it is time to think outside the box by (1) engaging with behavioral scientists (for example, to increase the self-confidence of doctors who keep prescribing broad-spectrum antibiotics even for viral infections) and (2) exploring solutions to find compensation systems to overcome purely economic interests (for example, to reduce the sale of antibiotics without prescriptions or the continued use of antibiotics as growth promoters).

Moreover, developing sophisticated tracking systems to monitor antimicrobial use (similar to blockchain approaches) could ensure immediate access when needed while flagging inappropriate use for rapid intervention. This approach would balance the critical need for access with responsible stewardship.

The fight against AMR is not just a medical challenge but also a test of our ability to act collectively in the face of a shared threat. Regarding microorganisms, AMR, and antimicrobial use, it is crucial to understand that there are no borders; the North–South divide is not as pronounced as in other healthcare aspects. Also, we cannot compartmentalize this issue because the environment links both human and animal sectors. Therefore, all solutions require us to think beyond national borders and sector-specific interests, embracing a truly global and interdisciplinary approach.

At this critical juncture, the resolutions made at the UN General Assembly will shape the future of global health security. Let us seize this moment to reaffirm our commitment to preserving the efficacy of antimicrobials for future generations. The time for decisive action is now; new antimicrobials will not be the solution if we do not first address the root causes of AMR.
